# Stressor richness intensifies productivity loss but mitigates biodiversity loss

**DOI:** 10.1002/ece3.8182

**Published:** 2021-10-12

**Authors:** Mark Holmes, Jurg Werner Spaak, Frederik De Laender

**Affiliations:** ^1^ Research Unit in Environmental and Evolutionary Biology, Namur Institute of Complex Systems, and the Institute of Life, Earth, and Environment University of Namur Namur Belgium

**Keywords:** community ecology, ecosystems, multiple stressors, theoretical ecology, traits

## Abstract

Ecosystems are subject to a multitude of anthropogenic environmental changes. Experimental research in the field of multiple stressors has typically involved varying the number of stressors, here termed stressor richness, but without controlling for total stressor intensity. Mistaking stressor intensity effects for stressor richness effects can misinform management decisions when there is a trade‐off between mitigating these two factors. We incorporate multiple stressors into three community models and show that, at a fixed total stressor intensity, increasing stressor richness aggravates joint stressor effects on ecosystem functioning, but reduces effects on species persistence and composition. In addition, stressor richness weakens the positive selection and negative complementarity effects on ecosystem function. We identify the among‐species variation of stressor effects on traits as a key determinant of the resulting community‐level stressor effects. Taken together, our results unravel the mechanisms linking multiple environmental changes to biodiversity and ecosystem function.

## Graphical abstract

Experimental research in the field of multiple stressors typically involves varying the number of stressors, but without controlling for total stressor intensity. We incorporate multiple stressors into community models and show that, at constant total stressor intensity, increasing stressor richness aggravates joint stressor effects on ecosystem functioning, but reduces effects on species persistence and community composition. We identify the among‐species variation of stressor effects on traits as a key determinant of the resulting community‐level stressor effects.

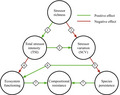

## 1 INTRODUCTION

Intensifying global human activities cause biological communities to experience increasingly numerous and severe environmental stressors. Stressor combinations have the potential to degrade ecosystem functioning and reduce biodiversity (Côté et al., 2016; Nogales et al., 2011). The ability of communities to resist these stressors will dictate the quality of the ecosystem services they provide in the future (Elmqvist et al., 2003). Thus, investigating the effect of multiple stressors in combination has become an intensely studied area of research (Orr et al., 2020).

Studies on the combined effect of multiple stressors typically rely on a standard factorial design: one measures first the effects of each stressor, and then of the stressor combinations. This approach allows testing for nonadditive effects of particular stressor combinations and especially targets the detection of synergistic effects, which can have severe consequences for ecosystem processes (Brennan & Collins, 2015; Côté et al., 2016; Crain et al., 2008; Darling & Côté, 2008; Rillig et al., 2019; Schäfer & Piggott, 2018). Nonadditive effects can be caused not only by direct stressor interactions (e.g., by affecting species’ physiology), but also by how species interact with each other and differentially respond to stressors (Baert, De Laender, et al., 2016; Baert, Janssen, et al., 2016; De Laender, 2018; Schäfer & Piggott, 2018; Thompson et al., 2018b). For instance, more species‐rich communities have higher complementarity and a higher likelihood of differential stressor sensitivities, which improve functional (total yield) and compositional (relative species abundances) resistance to stress (Baert, De Laender, et al., 2016; Baert, Janssen, et al., 2016; Craven et al., 2016; Isbell et al., 2015). Modeling studies taking such factors into account demonstrate that nonadditive stressor effects at the population level are expected to be the norm rather than the exception (De Laender, 2018; Piggott et al., 2015; Schäfer & Piggott, 2018; Thompson et al., 2018a).

A critical challenge associated with the standard factorial design is that the total stressor intensity experienced by the different species making up the community (TSI, i.e., the combined stressor effect experienced by all species) will often covary with the number of stressors, here termed “stressor richness” (De Laender, 2018; Schäfer & Piggott, 2018). Exposing a community to a higher number of stressors will generally lead to a higher TSI. Thus, it is difficult to disentangle the effects of stressor richness from the effects of the concomitant increase in TSI. For example, simultaneous exposure to both a herbicide and a moderate temperature increase (two stressors) can have the same negative effect on phytoplankton growth as exposure to a larger increase in temperature alone (single‐stressor) (Negri et al., 2011). Assessing the unique effect of stressor richness, as opposed to the joint effects of stressor richness and TSI, is important to optimize environmental management. Policymakers may face financial or practical constraints that cause trade‐offs between mitigation measures that reduce TSI and measures that reduce the number of environmental stressors (Côté et al., 2016). For example, restoring the hydromorphological features of a river (i.e., addressing a single stressor) yielded limited improvements in biodiversity overall because other impactful stressors (e.g., pollution) were not mitigated (i.e., TSI was not greatly reduced; Haase et al., 2013).

Here, we seek to tease apart the mechanisms of multiple stressor effects on biological communities in terms of effects on their functioning, diversity and composition. We present two hypotheses. Firstly, we hypothesize that, when stressor richness covaries with TSI, greater stressor richness would cause greater reductions in species richness, greater compositional change, and a greater loss of functioning (H1). We secondly hypothesize that these effects will differ when keeping TSI constant/fixed (H2).

We test these hypotheses in two ways. After analytical analyses of a simplified two‐species community model, we performed simulations using three community models of varying complexity (Lotka, 1978; MacArthur, 1970; Stomp et al., 2004; Table 1). We focused on competitive communities, a key module in real food webs. As there exist many different models simulating competition, we selected three that range from phenomenological to highly mechanistic. Using these models, we performed extensive in silico experiments in a factorial design to quantify the effect of stressor richness at fixed TSI (Equation 2 and Table 2), defining a stressor as a factor that affects species’ functional traits/growth parameters (De Laender, 2018; Litchman et al., 2015) (Equation 1). Our results robustly support both hypotheses. Specifically, all else being equal, increasing stressor richness weakens the effects on species persistence, community composition, and biodiversity effects on function, but aggravates effects on functioning. We explain the results based on the variability of stressor action among different species, coined the stressor coefficient of variation (SCV). Taken together, our results show how integrating multiple stressors into ecological theory fosters synthesis of community‐ and ecosystem‐level impacts.

**TABLE 1:** Species per capita growth rates by model and model parameters

Model	Per capita growth rate	Parameter		
Lotka–Volterra	$\mu_{i}‐\sum_{j=1}^{n}N_{j}\alpha_{\mathrm{ij}}$	$\mu_{i}$	$\theta_{i}$	Intrinsic growth rate of sp. *i*
		n		Number of species
		$N_{j}$		Population of sp. *j*
		$\alpha_{\mathrm{ij}}$		Effect of sp. *j* on sp. *i*
MacArthur	$\sum_{k=1}^{r}w_{i}c_{\mathrm{ik}}R_{k}‐m_{i}$	r		Number of resources
		$w_{i}$	$\theta_{i}$	Value of resources for sp. *i*
		$c_{\mathrm{ik}}$		Uptake of resource *k* by sp. *i*
		$R_{k}$		Resource *k* abundance
		$m_{i}$		Maintenance requirement of sp. *i*
Stomp	$\frac{\phi_{i}}{z}\int_{0}^{z}\gamma_{i}\left(z\right)dz‐l_{i}$	$\phi_{i}$	$\theta_{i}$	Sp. *i* photosynthetic efficiency
		Z		Water column depth
		$\gamma_{i}\left(z\right)$		Absorbed light by sp. *i* at depth *z*
		$l_{i}$		Specific loss rate of sp. *i*

Note

The traits/parameters that the stressors affect are indicated by $\theta_{i}$. For details on biological models, see Note S1.

**TABLE 2:** Factors manipulated in factorial simulation design

Factor	Factor levels
Total stressor intensity	10%, 50%, 90%, unfixed
Stressor richness	1, 2, 3, …, 20
Model	L‐V, M, S
Species richness	4, 8*, 16*
Stressor interactions	Absent, present

Note

L‐V, M, and S indicate Lotka–Volterra, MacArthur, and Stomp models, respectively. Initial species richnesses of 8 and 16 marked with an * were not used with the Stomp model.

## 2 METHODS

### 2.1 Models

We simulated competitive communities using three community models, which range from phenomenological to mechanistic (Table 1). More mechanistic models should more closely reflect specific real‐world scenarios, while more phenomenological models are more broadly applicable but also more simplified. No demographic stochasticity was present in any models: all processes were entirely deterministic.

In the Lotka–Volterra model (Lotka, 1978), the population size of a species depends on its intrinsic growth rate and on effects from inter‐ and intraspecific interactions. Species coexistence depends on the relative strength of intra‐ vs. interspecific interactions and the intrinsic growth rates.

MacArthur's consumer–resource model (MacArthur, 1970) describes a community of consumers competing for a number of resources whose dynamics are explicitly modeled. Resource densities increase logistically (S Equation 2), and surplus consumption is converted into population growth. Broadly speaking, species can coexist when they differ sufficiently in the resources they consume most, and do not have vastly different mortality rates (Chesson, 1990). Species uptake resources linearly (i.e., no density‐dependent effects), and resources are considered to be perfectly substitutable.

Finally, the most mechanistic model, described by Stomp et al. (2004), simulates a community of phytoplankton species competing for light. This is a relatively detailed model (Spaak & De Laender, 2021) whose model organisms, phytoplankton, are a vital part of aquatic/marine ecosystems. Phytoplankton face numerous anthropogenic stressors while they form the lowest trophic level upon which ecosystems depend, and provide 45% of atmospheric oxygen (Chavez et al., 2011; Häder & Gao, 2015). Species vary in their pigmentation and efficiency of converting light into growth, and are able to coexist by absorbing different parts of the incident light spectrum.

### 2.2 Incorporating stressors into the models

Stressors act directly on species’ functional traits (e.g., photosynthetic efficiency of phytoplankton; Litchman & Klausmeier, 2008) and thereby indirectly on population densities (Côté et al., 2016; Thompson et al., 2018a) and competitive outcomes (De Laender, 2018). We therefore consider stressors as factors that affect population growth but are not influenced by that population (e.g., temperature, pH, pollutants; Pásztor et al., 2016). The results of the simulations presented here may therefore not hold for environmental changes such as resource changes (De Laender, 2018), since resources are also consumed by populations (Meszéna et al., 2006).

Functional traits are represented by different parameters in the different models. Thus, the stressors affect model‐specific parameters, indicated by $\theta_{i}$ in Table 1. For Lotka–Volterra communities, stressors affected the species’ intrinsic growth rates, $\mu_{i}$. For MacArthur communities, stressors affected species‐specific resource values, $w_{i}$. Note that $w_{i}$ has been modified from its classical form: while it is traditionally a resource‐specific parameter, here it is species‐specific to enhance comparability to other models. For Stomp communities, stressors acted on the species’ photosynthetic efficiency, $\phi_{i}$. Reduction of $\phi_{i}$ by the action of stressors has been observed experimentally and in nature and is the mode of action of many herbicides to which phytoplankton are exposed (D’ors et al., 2016; Häder & Gao, 2015; Huertas et al., 2010; Kimmance et al., 2014).

The effects of *s* stressors on *n* species’ functional traits can be cast into an n×s matrix, E. An element of this matrix, $\varepsilon_{\mathrm{il}}$, is the multiplicative effect of stressor *l* on the functional trait of species *i*, $\theta_{i,0}$, that is, $\theta_{i}=\theta_{i,0}\prod_{l=1}^{s}\varepsilon_{\mathrm{il}}$, and is a number between 0 and 1. This effect is additive in the log space: $\log\left(\theta_{i}\right)=\log\left(\theta_{i,0}\right)+\sum_{l=1}^{s}\log(\varepsilon_{\mathrm{il}})$.

Interactive stressor effects on functional traits are species‐specific and covary with the noninteractive stressor effects, that is, stressors that have a greater effect are capable of having larger interactions (Vye et al., 2015). We quantify interactive effects between stressors *l*_1_ and *l*_2_ on the trait of species *i* by a factor $\eta_{il_{1}l_{2}}$:

(1) $$\text{log}\left(\theta_{i}\right)=\text{log}\left(\theta_{i,0}\right)+\underset{\text{Independent}\;\text{stressor}\;\text{effects}}{\underbrace{\sum_{l=1}^{s}\log(\varepsilon_{\mathrm{il}})}}+\underset{\text{Interactive stressor effects}}{\underbrace{\sum_{l_{1}=1}^{s}\sum_{l_{2}=1}^{s}\log\left(\varepsilon_{il_{1}}\right)\log\left(\varepsilon_{il_{2}}\right)\eta_{il_{1}l_{2}}}}.$$

Since stressor interactions act additively in the log space (Equation 1), this can produce both synergistic ( η<0) and antagonistic ( η>0) effects on $\theta_{i}$. For example, take the case of two stressors affecting a species *i*. Let $\varepsilon_{i1}=0.6$ and $\varepsilon_{i2}=0.8$ (effects of stressors 1 and 2 on species *i*, respectively). If $\eta_{il_{1}l_{2}}=0$, then there are no interactive effects and $\log\left(\theta_{i}\right)=\log\left(\theta_{i,0}\right)+\log\left(0.6\right)+\log\left(0.8\right)=\log\left(\theta_{i,0}\right)-0.22-0.097=\log\left(\theta_{i,0}\right)-0.32∴\theta_{i}=0.48\cdot \theta_{i,0}$. Thus, the parameter is now reduced to 48% of its original value $\theta_{i,0}$. If $\eta_{il_{1}l_{2}}>0$, then $\theta_{i}>0.48\cdot \theta_{i,0}$ and the interaction is antagonistic. Conversely, if $\eta_{il_{1}l_{2}}<0$, then $\theta_{i}<0.48\cdot \theta_{i,0}$ and the interaction is synergistic. Stressors do not interact with themselves (i.e., if $l_{1}=l_{2}$, $\eta_{il_{1}l_{2}}=0$).

We define total stressor intensity (TSI) as one minus the product of all stressor effects on all species traits ($1‐\prod_{i=1}^{n}\prod_{l=1}^{s}\varepsilon_{\mathrm{il}}$) such that for large effects of the individual stressors, this value approaches one (rather than zero, which mathematically shows strong stressor effects but is counterintuitive). Because we want to test for effects of stressor richness with both variable (Hypothesis 1, H1) and fixed/constant (Hypothesis 2, H2) TSI, we introduced the following scaling:

(2) $$\begin{array}{c} 1‐\varepsilon_{\mathrm{il}}^{\prime }=1‐\varepsilon_{\mathrm{il}}^{\frac{\log(d)}{\log\left(\prod_{i=1}^{n}\prod_{l=1}^{s}\varepsilon_{\mathrm{il}}\right)}}, \end{array}$$

where d=1‐TSI and takes some predefined value between 0 and 1. In this way, TSI is fixed such that any effects on communities subjected to different numbers of stressors are independent of TSI, allowing us to test H2. To test H1, we simply did not apply this scaling. Note that we will represent TSI as a percentage, for example, 90% stressor intensity (d=0.1). As an example, consider a two‐species (rows) two‐stressor (columns) matrix:

(3) $$\begin{array}{cc} E & =\left(\begin{array}{cc} 0.8 & 0.5\\ 0.4 & 0.7 \end{array}\right),\\ \text{TSI} & =1‐\prod_{i=1}^{2}\prod_{l=1}^{2}\varepsilon_{\mathrm{il}}\\ & =1‐(0.8\cdot 0.5\cdot 0.4\cdot 0.7)\\ & =1‐0.112=0.888=88.8\%. \end{array}$$

This means that species’ functional traits are, overall, affected by 88.8% across all stressors and species. Keeping the number of stressors as 2, but fixing TSI at 90%, is then done by rescaling the entries of E, using d=0.1 in Equation 2:

(4) $$\begin{array}{cc} E^{\prime } & ={\left(\begin{array}{cc} 0.8 & 0.5\\ 0.4 & 0.7 \end{array}\right)}^{\frac{\log(0.1)}{\log\left(\prod_{i=1}^{n}\prod_{l=1}^{s}\varepsilon_{\mathrm{il}}\right)}}\\ & =\left(\begin{array}{cc} 0.791 & 0.482\\ 0.381 & 0.687 \end{array}\right). \end{array}$$

One can now verify that the TSI of the rescaled matrix is 90%. The stressors still affect species in a proportionally similar manner, but the overall intensity is fixed.

### 2.3 Two‐species analyses

We first analyzed a simplified two‐species Lotka–Volterra model to analytically examine how stressor richness elicits ecological effects. If two species, 1 and 2, interact according to the Lotka–Volterra equation (Table 1) and do so symmetrically such that $\alpha_{12}=\alpha_{21}=\alpha$, they will be able to coexist in stressed conditions whenever:

(5) $$\begin{array}{c} \frac{\alpha }{\alpha_{11}}<\frac{\mu_{2}\prod_{l=1}^{s}\varepsilon_{2l}}{\mu_{1}\prod_{l=1}^{s}\varepsilon_{1l}}<\frac{\alpha_{22}}{\alpha }, \end{array}$$

where the products $\prod_{l=1}^{s}\varepsilon_{1l}$ and $\prod_{l=1}^{s}\varepsilon_{2l}$ represent the combined stressor effects on each species, as explained in Section 2.2. Note that we excluded stressor interactions for simplicity. For both species to persist under stressed conditions, the ratio $\rho =\prod_{l=1}^{s}\varepsilon_{2l}/\prod_{l=1}^{s}\varepsilon_{1l}$ should not differ too much from 1. Larger deviations from 1 indicate that one species is more strongly affected by the stressors than the other and there is a higher risk of species loss (Chesson, 2000). We therefore asked how this ratio changes as stressor richness increases, and how this change differs for fixed vs. variable TSI. As the average ratio will not change (stressors are generated randomly), we instead looked at the variation around the average.

Finally, we apply the simplified two‐species case to explore the impact on biodiversity effects (selection and complementarity) on ecosystem functioning (Loreau & Hector, 2001). These effects characterize communities by whether their community yield is governed primarily by high productivity of competitive species (selection effect) or by niche partitioning (complementarity effect). The sum of these biodiversity effects is the net biodiversity effect (ΔY), which is the difference between the expected total yield (the sum of the monoculture yields multiplied by the expected relative yield, here 0.5) and the observed total yield in the community.

(6) $$\begin{array}{c} \Delta Y=\bar{\Delta RY}\;\bar{M}+n\text{cov}\left(\Delta RY,M\right), \end{array}$$

where ΔRY is the deviation from the species’ expected relative yields, *M* is the sum of the monoculture yields, and *n* is the number of species (Loreau & Hector, 2001). $\bar{\Delta RY}\;\bar{M}$ is the complementarity effect, and ncov(ΔRY,M) is the selection effect. Both effects were scaled to be comparable between different communities by dividing them by ΔY.

### 2.4 Simulations

Next, using all three models, we adopted a factorial design including five factors, simulating 1000 communities for each factor combination: total stressor intensity (unfixed or fixed at values in Table 2), stressor richness, community model, initial species richness, and presence of trait‐level stressor interactions (i.e., stressor interactions that have a direct effect on θ; Table 2). The design was fully factorial with the exception that we did not vary initial species richness for the Stomp model, as coexistence of more than four species on light spectrum differentiation alone proved impossible (Spaak & De Laender, 2021). We set the upper limit of stressor richness to 20, similar to the maximum number of stressors reported in analyses of field data (Côté et al., 2016; Halpern et al., 2008). In total, these factors amounted to 1120 combinations, that is, 1,120,000 simulations.

We generated communities of a set number of species that coexisted in the absence of stress. To do so, we first randomly generated parameters for each model, described in Table 1, within ranges that were likely to result in stable coexistence (Table S1). The focal parameters from the three models ($\theta_{i}$ in Table 1) were all sampled from the same type of distribution to aid comparability between models. For simulations with the Stomp model, $\phi_{i}$ (photosynthetic efficiency) values were sampled from a log‐normal distribution, spanning a realistic range (ϕ is strongly linked to cell size, which follows this distribution in nature, Langdon, 1988; Stomp et al., 2004; Finkel et al., 2010; Ryabov et al., 2021; Spaak & De Laender, 2021). Consequently, the focal parameters for the other models, $\mu_{i}$ and $w_{i}$ for Lotka–Volterra and MacArthur communities, respectively, were also sampled from a log‐normal distribution. Other parameter generation settings are detailed in the Appendix S1.

We only considered communities where all species in the community were present at ≥1% of their carrying capacity. Any species whose population was below this threshold were considered extinct, and the community was not used, to ensure that all species were present in an ecologically meaningful way. Population densities at equilibrium were computed by solving the differential equations using Broyden's method with the R package “nleqslv” (Broyden, 1965; Hasselman, 2017). For each community, we then generated a species stressor matrix E and determined the new stressed community equilibrium using the same method. ε values were sampled from a beta distribution, such that they varied between 0 and 1, with less intense stressors being more common: $\varepsilon ∼\text{Beta}\left(\alpha =6.5,\beta =0.25\right)$. Because stressors affected species randomly, there were no overall patterns of cotolerances (Vinebrooke et al., 2004). When present, stressor interactions, η, were sampled from a normal distribution with a mean of zero to have an additive effect on average, with small interactions being more frequent: $\eta ∼N\left(\mu =0,\sigma =1\right)$. When TSI was fixed, we rescaled E according to Equation 2 (the exact value of *d* used is arbitrary).

To assess the effects of stress on the community, we measured ecosystem function as the total abundance (yield) of all surviving species. We also measured species persistence as the number of surviving species. We compared these two metrics to function and persistence in the absence of stressors. Additionally, we also measured compositional resistance, which indicates how similar in composition the stressed community is to the unstressed community, by using the Bray–Curtis similarity index (Bray & Curtis, 1957). Finally, we again measured the biodiversity effects introduced earlier: selection and complementarity.

We anticipated that the driving force behind species loss was likely to be inequality in how much different species are affected by stressors (De Laender, 2018). We quantified this by measuring the stressor coefficient of variation (SCV), the coefficient of variation of the combined stressor effects on each species, that is, $\text{CV}\left(\prod_{l=1}^{s}\varepsilon_{\mathrm{il}}\right)$.

## 3 RESULTS

### 3.1 Analyses of simple two‐species communities

**FIGURE 1:**
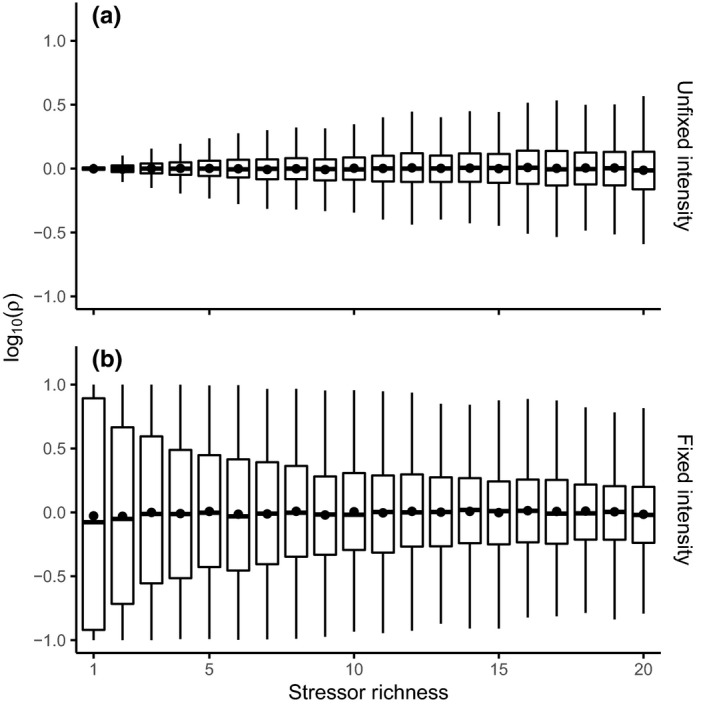
Stressor richness effects on two‐species stressor effect ratios, *ρ*, for variable (a) and fixed (b) stressor intensities, based on 1000 iterations for each box. *ρ*, presented on a log‐scale, is the total stressor effect on species 1 divided by the total stressor effect on species 2 indicating the difference in how affected each species is by stressor effects. Points indicate mean values

The two‐species communities analyzed in this section were generated using the Lotka–Volterra model only. When TSI is unfixed and thus covaries with stressor richness (Figure 1a), adding more stressors increases the likelihood of obtaining a ratio *ρ* that is too different from 1 for both species to coexist. The exact threshold value for coexistence is irrelevant: If such a value exists, a greater variance of *ρ* will lead to more cases of species loss. When TSI is fixed at a certain value (Figure 1b), the variance of *ρ* now decreases with increasing stressor richness: it becomes less likely to obtain a ratio *ρ* sufficiently far from 1 to cause an extinction. Recall that it is the TSI, $1‐\prod_{i=1}^{n}\prod_{l=1}^{s}\varepsilon_{\mathrm{il}}$, across all species that is fixed (Section 2.2), and thus, it is still possible that one species is notably more affected. Now as stressor richness increases, due to sample size effects it becomes increasingly unlikely for species to greatly vary in the stressor effects which they experience.

We also applied the simple two‐species case to analytically explore multiple‐stressor effects on community functioning. Here, the total yield observed under stressed conditions ($Y_{o}$) divided by the total yield observed in pristine conditions ($Y_{\text{op}}$) equals (setting $\alpha_{11}=\alpha_{22}=1$ and $\mu_{1}=\mu_{2}=1$, Supplements):

(7) $$\begin{array}{c} \begin{array}{c} \frac{Y_{o}}{Y_{\text{op}}}=\frac{\prod_{l=1}^{s}\varepsilon_{1l}+\prod_{l=1}^{s}\varepsilon_{2l}}{2}. \end{array} \end{array}$$

Effects on functioning ($Y_{o}$) are, unlike effects on persistence, driven by the TSI and not by the ratio *ρ*. When TSI is unfixed, and thus increases with stressor richness, adding more stressors will reduce both products $\prod_{l=1}^{s}\varepsilon_{\mathrm{il}}$ (as $0<\varepsilon_{\mathrm{il}}<1$), thus decreasing functioning ($Y_{o}/Y_{\text{op}}$). At a fixed TSI, few stressors will permit species to differ substantially in the stressor effect they experience: only the *product* of all stressor effects across all species is fixed (Section 2.2). This leads to substantial variation in the *sum* of these effects, which features in Equation 7. However, as more stressors are added, this variation decreases: every species now “samples” a sufficiently large number of stressors for $\varepsilon_{\mathrm{il}}$ to stabilize with stressor richness and to be comparable across species.

As the ratio of stressor effects between the two species, *ρ*, affects the stress‐induced yield reduction $Y_{o}/Y_{\text{op}}$, this ratio also predicts complementarity *C* (scaled by the net biodiversity effect ΔY) (Supplements):

(8) $$\begin{array}{c} \frac{C}{\Delta Y}=\frac{\alpha \left(\alpha ‐\rho ‐\frac{1}{\rho }\right)+1}{{\left(\alpha ‐1\right)}^{2}}, \end{array}$$

and the selection effect *S* (scaled by ΔY):

(9) $$\begin{array}{c} \frac{S}{\Delta Y}=\frac{\alpha \left(\frac{1}{\rho }‐2+\rho \right)}{{\left(\alpha ‐1\right)}^{2}}. \end{array}$$

As explained above and shown in Figure 1, when TSI is unfixed, increasing stressor richness causes the ratio *ρ* to deviate more frequently from 1 (Figure 1). Equations 8 and 9 (visualized in Figure S2) show that greater deviations from 1 lead to lower complementarity and higher selection effects. Conversely, when TSI is fixed, stressor richness leads to smaller deviations from ρ=1 (Figure S2), leading to higher complementarity and lower selection effects. These changes are more pronounced when species interactions (*α*) are stronger. These analytical results highlight the importance of the among‐species variation of stressor effects, which is what we now report on for more realistic and species‐rich communities.

### 3.2 Stressor effects on multispecies communities

**FIGURE 2:**
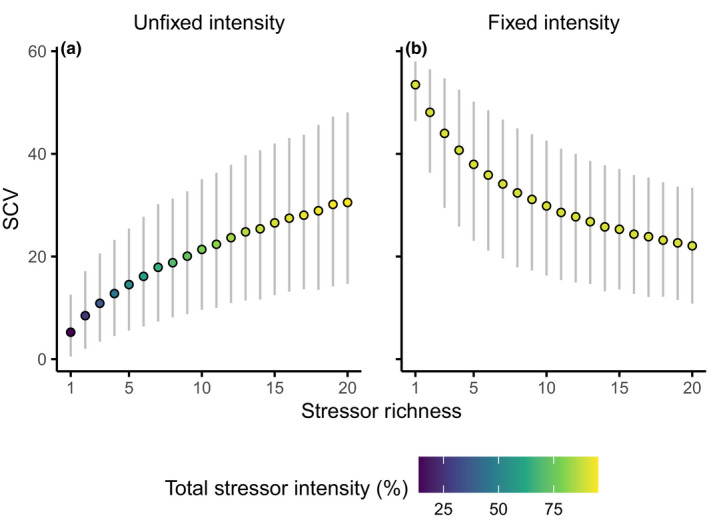
Effect of stressor richness on stressor coefficient of variation (SCV, i.e., the coefficient of variation of the per‐species stressor intensity). Panels indicate variable (a) and fixed (b) total stressor intensity (TSI, the product of all stressor e effects on all species). Dots indicate mean values, and error bars show the 10th–90th percentile range of the 3000 simulations per stressor setting, totaling 180,000 simulations

When TSI was unfixed, the coefficient of variation of the among‐species stressor effects (SCV, i.e., the coefficient of variation of the row products of E) increased with stressor richness (Figure 2a). When TSI was fixed, SCV decreased with stressor richness (Figure 2b), with a much stronger decline present at higher TSI (Figure S3). Without keeping TSI fixed, increasing stressor richness inflated differences among the combined stressor effects experienced by species, but when TSI was fixed, increasing stressor richness reduced such differences.

**FIGURE 3:**
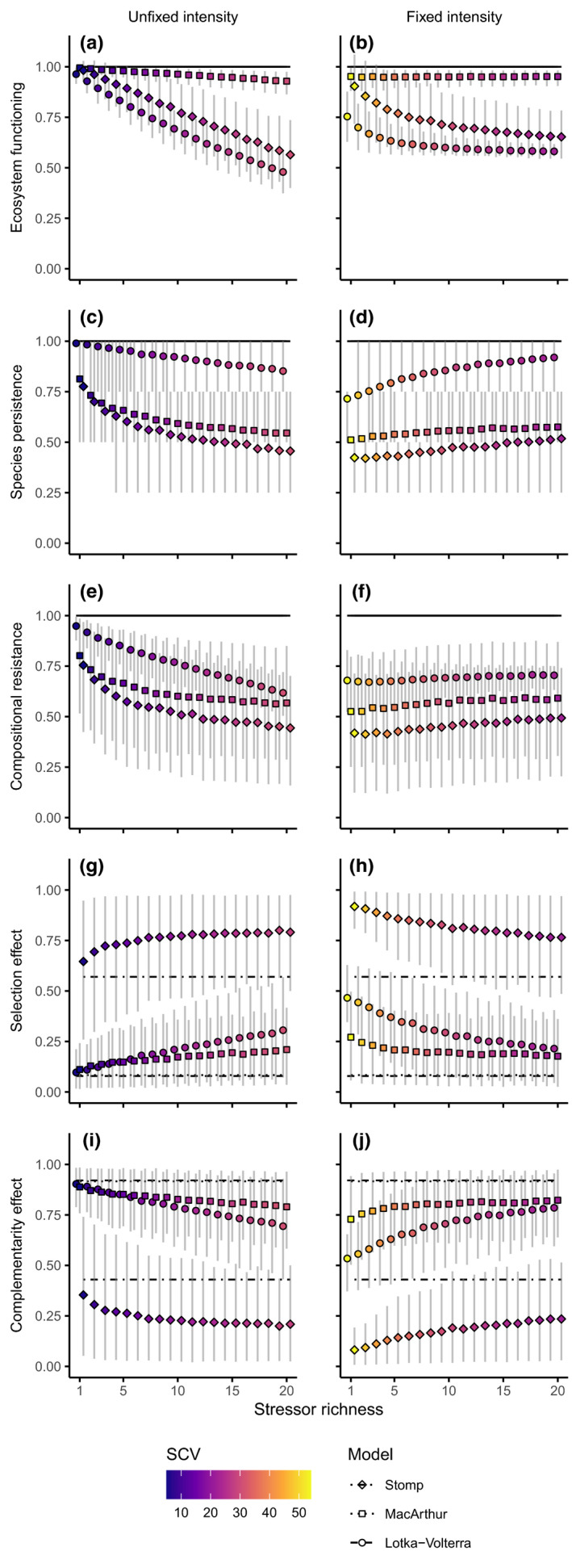
Stressor richness and stressor coefficient of variation (SCV, i.e., the coefficient of variation of the per‐species stressor intensity) effects on community metrics (ecosystem functioning, species persistence, and compositional resistance) and biodiversity effects (selection and complementarity). These metrics are shown for variable (a, c, e, g, i) and fixed (b, d, f, h, j) total stressor intensity. Horizontal dashed line shows the unstressed community value. Dots indicate mean values, and error bars show the 10th–90th percentile range of the 1000 simulations per simulation setting, totaling 180,000 simulations

For visual clarity, Figures 2 and 3 show only the results of the four‐species simulations only, without stressor interactions (η=0 in Equation 1), as varying these factors did not greatly alter the main findings (Figure S2). Increasing the initial species richness slightly improved overall resistance to stress, and the presence of stressor interactions had no notable effect overall. Additionally, to contrast the effects of controlling TSI, we show only simulations where TSI was unfixed or fixed at 90%. This provides the greatest contrast to demonstrate the effects most clearly.

When TSI was unfixed and allowed to vary with stressor richness (Figure 3a,c,e,g,i), higher stressor richness strongly reduced ecosystem functioning, species persistence, and compositional resistance. However, with fixed TSI (Figure 3b,d,f,h,j), a different response emerged: stressor richness reduced the negative stressor effect on ecosystem functioning, and improved species persistence and compositional resistance. When TSI was unfixed, complementarity decreased with stressor richness, while selection increased. Exactly the opposite patterns emerged when TSI was fixed.

Among‐species variation of stressor effects (SCV) covaried with the reported effects on function, persistence, composition, and both biodiversity effects. Low stressor richness led to large differences among species (high SCV), which limited effects on functioning but worsened effects on persistence, composition, and biodiversity effects.

All simulation results qualitatively matched the analytical results obtained for the simple two‐species model. That is, effects on functioning indeed stabilized with stressor richness at high stressor richness, and among‐species differences of stressor effects (*ρ* in the two‐species case; here, SCV) explained stressor impacts on persistence, composition, and biodiversity effects. Thus, we conclude that the mechanisms identified with the simple model explain the results in the more realistic models and scenarios.

## 4 DISCUSSION

We obtained theoretical results for the effects of multiple stressors on ecosystem functioning (Figure 3a,b), species persistence (Figure 3c,d), community composition (Figure 3e,f), and two biodiversity effects (g–j, Figure S2; Loreau & Hector, 2001). Together, these results show that the effect of stressor richness causes negative ecological effects (confirming H1), but not when total stressor intensity (TSI) is fixed (confirming H2). These results mechanistically underpin the importance of relative sensitivities, which are quantified through the ratio *ρ* in the case of species pairs, or through the stressor coefficient of variation (SCV) in the case of multiple species. While these results confirm previous results for single stressors (Baert et al., 2018), they demonstrate that information on which stressor affects which species is not needed to predict ecological change, only the total effect per species is necessary. We expect these results to be general for stressors with a multiplicative effect on model parameters (e.g., temperature; Uszko et al., 2017).

The results highlight the importance of separating the components of multiple stressor effects into mechanistic measures (stressor richness, TSI, and SCV) to better understand the link between multivariate environmental change and ecological change. When TSI is not fixed, the effects of stressor richness reflect published empirical and modeling results (Brennan & Collins, 2015; Garnier et al., 2017; Rillig et al., 2019; Thompson et al., 2018b), confirming H1. However, increasing stressor richness alone, while keeping TSI fixed, yields different and initially counterintuitive community‐ and ecosystem‐level effects, confirming H2. Stressor richness decreased ecosystem functioning (total population/biomass yield), albeit modestly, and increased compositional resistance to stress (the degree to which stress changed community composition; Figure 3). Degradation of ecosystems is mainly driven by increasing TSI, while changes in community composition are more due to variation in among‐species stressor effects. Both factors may vary with stressor richness, but it is important to consider their effects separately.

Prior studies have noted the positive impact of stressors on the selection effect (negative impact on complementarity; Baert et al., 2018), which is reflected in our results (Figure 3g,i). However, when fixing TSI, we obtained the opposite result. Complementarity between species is maintained if stressors act equally at a fixed intensity. Thus, without controlling TSI, species loss will result in greater loss of function when more stressors are present. Conversely, when keeping TSI constant, species loss will affect function less when more stressors are present.

SCV had notable effects on all metrics (Figure 3), indicating that capturing similarities and differences in stressor effects among species can be used to predict community‐level effects. This result is somewhat surprising as the effects of environmental change on a species will also depend on their ecology (Arnoldi et al., 2019; Baert et al., 2017; De Laender et al., 2016; Hodgson et al., 2017). The matrices E from which SCV is computed do not contain such information. The success of this metric to predict ecological impact may be explained by the focus of the present study on communities of relatively comparable ecology (competitive communities).

As data on environmental effects on species traits become increasingly available (Dengler et al., 2011; Edwards et al., 2013; Iversen et al., 2017; Kattge et al., 2011), applying the presented theory to forecast ecological change becomes increasingly feasible. Ideally, the biological responses (e.g., of intrinsic growth rate) of *n* species to *s* stressors (e.g., pollutants, temperature) are available as *ns* functions *f* that return the response for each species stressor combination, using the *s* stressor values as an input (Schäfer & Piggott, 2018). The product of all *ns* responses, at some combination of *s* stressor values, is the TSI, $1‐\prod_{i=1}^{n}\prod_{l=1}^{s}\varepsilon_{\mathrm{il}}$. Experiments could then measure how TSI affects various ecological variables. Controlling TSI while varying SCV is more challenging and will depend on the shapes of the aforementioned functions *f*. Experiments and analyses of monitoring data based on the basic principles laid out in the present paper are needed to help connect observed environmental and biodiversity change (Bowler et al., 2020; Daskalova et al., 2020).

Interactive effects among stressors are of concern in global change ecology (Orr et al., 2020) and can manifest at multiple organizational levels. Including trait‐level stressor interactions did not qualitatively influence our results (Figures S4–S6). However, this observation does not imply a limited influence of trait‐level stressor interactions on the prevalence of stressor interactions at higher organizational levels. That is not only because we did not explicitly test for such influence, but also because we did not have data to parameterize trait‐level stressor interactions. We can therefore not assert that this parameter setting was realistic. For example, we assumed that stressor interactions at a trait level were as likely to be synergistic or antagonistic.

**FIGURE 4:**
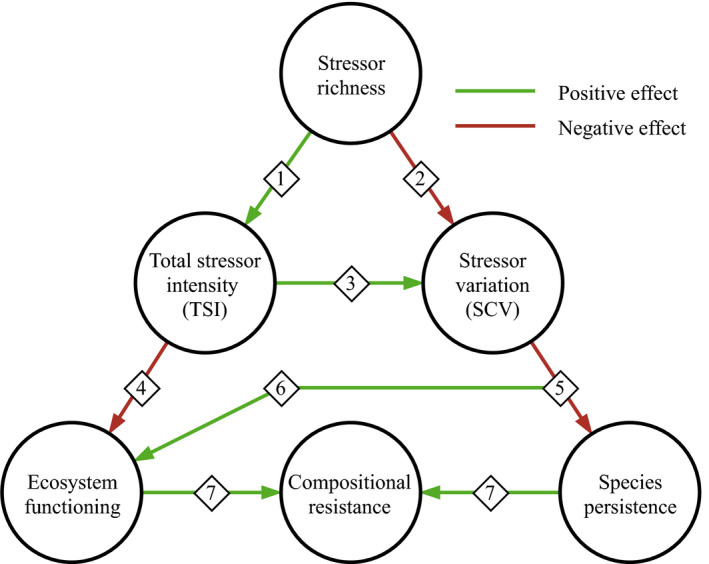
Main relationships between components of stressor action and community metrics. (1) Increased stressor richness causes greater total stress intensity; (2) increased stressor richness causes species to be affected more similarly; (3) greater total stressor intensity (TSI) increases the mean stressor effect (<1), increasing SCV; (4) greater stressor intensity decreases ecosystem functioning; (5) unequal stressor effects cause competitive exclusion; (6) remaining species experience competitive release; and (7) species persistence and ecosystem functioning translate to increased compositional resistance

Figure 4 shows conceptually the relationships between stressor richness and the studied community metrics. By separating stressor richness effects into TSI and SCV, we can better understand the links between environmental and ecological change. Although these mechanisms are quantified by our mathematical results, they also permit intuitive understanding. When TSI was fixed (Figure 2b,d,f,h,j), low stressor richness resulted in high SCV, creating differences among species sensitivities (large deviations from ρ=1), allowing for compensation by less sensitive species through competitive release, and therefore smaller effects on ecosystem functioning (Figure 2b). Higher stressor richness results in low SCV (i.e., stressors affect all species similarly; Figure 2), such that no species is able to compensate for loss of function (Figure 3b,e). At the same time, this reduces the likelihood of species extinction: the community is more similar to its pre‐stress composition (Figure 3).

The described methodology makes several assumptions, which should be considered when interpreting the results. Firstly, stressor action does not vary over time, making them press, rather than pulse perturbations. Therefore, it is unclear whether our results give insight into how community dynamics would respond to environmental variation over time (Arnoldi et al., 2019; Radchuk et al., 2019). We expect this dynamic behavior to be especially important in case of temporal variation of stressor richness and intensity. For example, if a first stressor causes the community to switch to priority effects by changing species interactions (Grainger et al., 2019), the response to a second stressor will be different than when it had occurred before the first stressor (Brooks & Crowe, 2019). Additionally, we consider only stressors, which negatively affect growth, while many environmental factors in nature may increase population growth rate in certain cases, for example, unimodal temperature effects on photosynthesis (Häder & Gao, 2015) or attack rates (Uszko et al., 2017). While including such stressors would certainly change how ecosystem function compares with unstressed ecosystem function, the same mechanisms linking stressor richness and diversity to species richness listed above would apply (Thompson et al., 2018b). Just like stressors reducing growth, stressors increasing growth create differences among species that can result in exclusion of those species experiencing lower increases in growth (Baert, De Laender, et al., 2016; Baert, Janssen, et al., 2016; Figure 1). Finally, because the matrix E was constructed randomly, we did not include any systematic patterns of cotolerance, which can influence the likelihood of species loss and can have important consequences on stressor‐induced community change (Vinebrooke et al., 2004).

Here, we present new insights into how stressor richness affects community structure and function by severing the normally associated influence of TSI. The effects of increasing stressor richness were less pronounced when the TSI was fixed. The most notable difference between fixed and unfixed stressor action, however, was the positive influence of stressor richness on species diversity and community composition. This is a potentially encouraging finding: limiting the total intensity of stressors helps maintain species diversity. This improved diversity can provide better resistance to additional stressors and provide functional redundancy in the community, ensuring that ecosystem services continue to function. The approach demonstrated here offers future avenues of possible research that would expand on these results. Firstly, expanding to other types of species interactions (e.g., trophic, mutualist), is an important next step. As the community‐level impacts of stressors differ depending on the type of species interactions, we may expect that SCV will also have different effects depending on species interaction type (Thompson et al., 2018b; Zhao et al., 2019). Secondly, allowing stressors to directly affect species interactions, which is commonplace in real ecosystems, would also be beneficial (Daugaard et al., 2019; Valiente‐Banuet et al., 2015). More generalized theoretical approaches, such as those presented here, would allow insights into the mechanisms and rules governing multiple stressor effects.

## CONFLICT OF INTEREST

The authors declare no conflicts of interest.

## AUTHOR CONTRIBUTIONS

**Mark Holmes:** Conceptualization (equal); Formal analysis (lead); Investigation (equal); Methodology (equal); Visualization (equal); Writing‐original draft (lead); Writing‐review & editing (equal). **Jurg Werner Spaak:** Formal analysis (equal); Investigation (equal); Methodology (equal); Visualization (equal); Writing‐original draft (equal); Writing‐review & editing (equal). **Frederik De Laender:** Conceptualization (equal); Funding acquisition (lead); Investigation (equal); Methodology (equal); Project administration (equal); Supervision (lead); Visualization (equal); Writing‐original draft (equal); Writing‐review & editing (equal).

## Supporting information

Appendix S1

## Data Availability

Code for generating communities and stressors and calculating stressor diversity and community metrics as shown in the article is publicly available at: github.com/markjholmes/stressor_richness. Simulations were performed using R version 4.0.3.
