# Supplementary material for: Stressor richness intensifies productivity loss but mitigates biodiversity loss
Source: Ecol Evol. 2021 Oct 12;11(21):14977–87. doi: 10.1002/ece3.8182 (PMC8571636; doi:10.1002/ece3.8182)
Supplement: Supplementary file 1 — Appendix S1 [file ECE3-11-14977-s001.pdf]

Stressor richness intensifies productivity loss but mitigates biodiversity loss: Supplementary information

Mark Holmes, Jurg W. Spaak, Frederik De Laender

Model details

MacArthur (MacArthur, 1970) and Stomp (Stomp et al., 2004) model formulations are simplified in the main text, and presented here are the full formulations used in simulations. Lotka-Volterra model details are restated for easier understanding of the two-species community analyses.

Lotka-Volterra

$$\frac{1}{N_i}\frac{\mathrm{d}N_i}{\mathrm{d}t} = \mu_i - \sum_{j=1}^{n} N_j \alpha_{ij} \tag{1}$$

The per-capita population growth rate of a species i depends on its intrinsic growth rate, μ_i, and on effects from inter- and intra-specific interactions α_{ij}.

MacArthur

$$\frac{1}{N_i}\frac{\mathrm{d}N_i}{\mathrm{d}t} = b_i \left(\sum_{l=1}^{r} c_{il} w_i R_l - m_i \right) \tag{2}$$

While consumer growth depends on resource availability, resources are renewed at a constant rate, up to a maximum resource saturation (i.e. logistically). The dynamics of resource l is described as such:

$$\frac{1}{R_l}\frac{\mathrm{d}R_l}{\mathrm{d}t} = s_l \left(1 - \frac{R_l}{K_l} \right) - \sum_{i=1}^{n} c_{il} N_i \tag{3}$$

Resource maximum saturation (K_l) and resource supply rates (s_l) were kept constant between simulation settings. Species consumption rates of resources (c_{il}) were defined as species-specific random normal distributions whose means and standard deviations were randomly generated using a random uniform distribution. In this way, all species had different preferred resources and niche widths. The species maintenance requirements (m_i) and the resource value (w_i) were generated using a random uniform distribution, and the factor which converts excess resources into growth (b_i) was kept constant, as it does not affect the equilibrium position.

Note that w_i has been modified from its classical form: while traditionally it is a property of each resource, dictating that resources value for all species, here we use it as a species-specific value for all resources, implying that all resources are equally important for each species' growth, but that they are not equally important for different species. This modification is necessary to compare the effects of stressors on species: if it were kept as w_l, the stressors would effectively act on the resources rather than species. As the number of resources did not always equal the number of species, this would have made direct comparison to other models more difficult. The modification does not change the overall conclusions.

Stomp

$$\frac{1}{N_i}\frac{\mathrm{d}N_i}{\mathrm{d}t} = \frac{\phi_i}{z_m} \int_0^{z_m} \gamma_i(z)\,\mathrm{d}z - L_i \tag{4}$$

$$\gamma_i(z) = \int_{400}^{700} I(\lambda, z) k_i(\lambda)\,\mathrm{d}\lambda \tag{5}$$

$$I(\lambda, z) = I_{in}(\lambda) \exp\left(-\sum_{i=1}^{n} k_i(\lambda) N_i z - k_{bg}(\lambda) z \right) \tag{6}$$

ϕ_i (photosynthetic efficiency) and k_i (species absorption spectra) values were randomly generated to resemble those of real phytoplankton taxonomic groups (Spaak and De Laender, 2021). The underwater light spectrum ($I(\lambda, z)$) is dependent on the incident light spectrum ($I_{in}(\lambda)$) and background light absorption spectrum ($k_{bg}(\lambda)$). These parameters, as well as z_m (mixing depth of the water column), and L_i (loss rate of the species) were taken from Stomp (2007) (Stomp et al., 2007).

Parameter values for factorial results

Table 1: Parameter generation for different models. $\mathcal{N}(a, b)$ indicates a normal distribution with a mean of a and a standard deviation of b. Lognormal(a, b) indicates a log-normal distribution with a mean of a and a standard deviation of b (both on a log scale). U(c, d) indicates a uniform distribution with a minimum of c and a maximum of d.

Model	Parameter	Generation
Lotka-Volterra	μ_i	Lognormal$(\ln(2.5), \ln(1.25)) \cdot 2$
	α_{ij}	$\mathcal{N}(2.5e^{-4}, 2.5e^{-5})$
	α_{ii}	$\mathcal{N}(5e^{-3}, 5e^{-4})$
MacArthur	b_i	1
	w_i	Lognormal$(\ln(2.5), \ln(1.25)) \cdot 10$
	c_{ik}	see section MacArthur
	m_i	$\mathcal{U}(1, 3)$
Stomp	ϕ_i	Lognormal$(\ln(2.5), \ln(1.25)) \cdot 1e^6$
	z_m	50
	I	From real values (Stomp et al., 2007)
	k_i	From real values (Stomp et al., 2007)
	k_{bg}	From real values (Stomp et al., 2007)
	l_i	$3e^{-3}$

Simple two-species communities

In a competitive Lotka-Volterra model of two species, 1 and 2, they will be able to coexist whenever:

$$\frac{a}{a_{11}} < \frac{\mu_2 \prod_l^s \varepsilon_{2l}}{\mu_1 \prod \varepsilon_{1l}} < \frac{a_{22}}{a}, \tag{7}$$

where all symbols are as in the manuscript and ε_{1l} is the effects of stressor l on species 1 (same for 2). So, given that the two species coexist in absence of the stressors, this means that the ratio $\rho = \prod_l^s \varepsilon_{2l} / \prod_l^s \varepsilon_{1l}$ should not be too different from 1 for both species to persist. Conversely, greater deviations from 1 indicate a larger risk of losing one of the species.

Two-species results

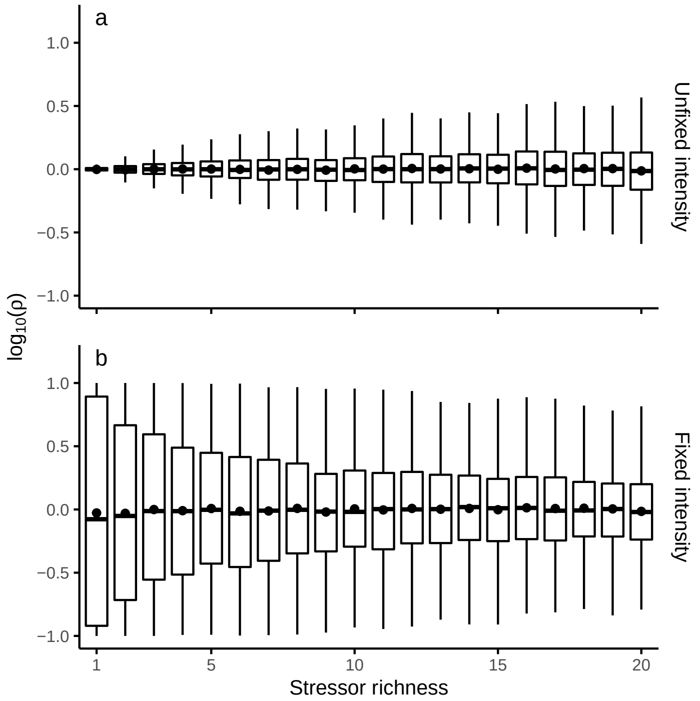

Figure S1: The log of the quotient ρ vs. stressor richness.

When we allow ε to vary, we find that the average ratio (based on 1000 iterations) does not change, but the variance does: it gets increasingly likely to obtain a ratio ρ that is "too different" from 1 for both species to coexist (Figure S1a). Note that we did not specify from which value onwards we will lose species, as this is irrelevant. The point is that there exists such a value, and the greater the variance of the ratio ρ, the more cases we will have among our 1000 iterations in which a species gets lost.

This result was to be expected, since for many distributions, the product of s random variables (where s = stressor richness) will have a variance that is greater than that of the individual variables. Thus: this explains the main result of stressor richness on species richness in the paper. Adding more stressors will inflate the variance of the numerator and denominator of the quotient ρ, and therefore of the quotient itself.

When ε *is* fixed, we find that the average ratio (based on 1000 iterations) again does not change, but again the variance does (Figure S1b). However, the variance now drops as stressors are added. It gets less likely to obtain a ratio ρ that is "too different" from 1 for both species to coexist (Figure S1) as we add stressors.

Again, this result was expected. To understand this, recall that we are controlling the *community-level* stress intensity. Thus, it is perfectly possible that one species is strongly impacted by our combination of stressors, while the other is not. The only thing that matters is that the total effect across species is always the same. As we add more stressors, it gets increasingly unlikely to have one species impacted more than the other. This is simply a sample size effect: the more stressors, the more effects ε you need to sample, and so the more likely the resulting product will be close to the expected product. Adding more stressors will thus shrink the variance of the numerator and denominator of the quotient ρ, and therefore of the quotient itself.

Ecosystem function

For a competitive Lotka-Volterra model of two species 1 and 2, taking $\alpha_{11} = \alpha_{22} = 1$ and $\alpha_{12} = \alpha_{21} = \alpha$, total biomass yield Y_o is the sum of the yield of species 1 (Y_{o1}) and that of species 2 (Y_{o2}):

$$
\begin{aligned}
Y_{o1} &= \frac{1}{1-\alpha^2}\left(\mu_1 \prod_l^s \varepsilon_{1l} - \alpha\mu_2 \prod_l^s \varepsilon_{2l}\right) \\
Y_{o2} &= \frac{1}{1-\alpha^2}\left(\mu_2 \prod_l^s \varepsilon_{2l} - \alpha\mu_1 \prod_l^s \varepsilon_{1l}\right) \\
Y_o &= \sum_i^2 Y_{oi} = \frac{1}{1-\alpha^2}\left(\mu_1 \prod_l^s \varepsilon_{1l} + \mu_2 \prod_l^s \varepsilon_{2l}\right)(1-\alpha) \\
&= \frac{1}{1+\alpha}\left(\mu_1 \prod_l^s \varepsilon_{1l} + \mu_2 \prod_l^s \varepsilon_{2l}\right)
\end{aligned}
\tag{8}
$$

Setting $\mu_1 = \mu_2 = 1$ for simplicity leads to $\frac{Y_o}{Y_{op}} = \frac{\prod_l^s \varepsilon_{1l} + \prod_l^s \varepsilon_{2l}}{2}$, where the subscript p refers to yield under pristine conditions. This expression makes clear that, unlike the results for coexistence, effects on function are not determined by the ratio of stressor effects but by their sum.

Selection and complementarity

The complementarity effect is defined as $N\overline{RY}\ \overline{M}$, where N is the species richness, $\overline{RY}$ is the mean deviation from expected relative yield of all species in the mixture, $\overline{M}$ is the mean species yield in monoculture. The selection effect is defined as $N\mathrm{cov}(\Delta Y, M)$, where $\mathrm{cov}(\Delta Y, M)$ is the covariance of deviation from expected relative yield of each species and the yield of each species in monoculture.

We can find an explicit expression for both biodiversity effects in the case of a competitive Lotka-Volterra model of two species 1 and 2. For such a model, taking $\alpha_{11} = \alpha_{22} = 1$ and $\alpha_{12} = \alpha_{21} = \alpha$ results in:

$$
\begin{aligned}
M_1 &= \mu_1 \prod_l^s \varepsilon_{1l} \\
M_2 &= \mu_2 \prod_l^s \varepsilon_{2l} \\
Y_{o1} &= \frac{1}{1-\alpha^2}\left(\mu_1 \prod_l^s \varepsilon_{1l} - \alpha\mu_2 \prod_l^s \varepsilon_{2l}\right) \\
Y_{o2} &= \frac{1}{1-\alpha^2}\left(\mu_2 \prod_l^s \varepsilon_{2l} - \alpha\mu_1 \prod_l^s \varepsilon_{1l}\right) \\
Y_o &= \frac{1}{1+\alpha}\left(\mu_1 \prod_l^s \varepsilon_{1l} + \mu_2 \prod_l^s \varepsilon_{2l}\right) \\
RY_{e1} &= RY_{e2} = 1/2 \\
RY_{o1} &= \frac{1}{1-\alpha^2}\left(1 - \alpha\frac{\mu_2 \prod_l^s \varepsilon_{2l}}{\mu_1 \prod_l^s \varepsilon_{1l}}\right) \\
RY_{o2} &= \frac{1}{1-\alpha^2}\left(1 - \alpha\frac{\mu_1 \prod_l^s \varepsilon_{1l}}{\mu_2 \prod_l^s \varepsilon_{2l}}\right) \\
\Delta RY_1 &= RY_{o1} - 1/2 \\
\Delta RY_2 &= RY_{o2} - 1/2,
\end{aligned}
\tag{9}
$$

where all symbols are as described in the methods. The monoculture equilibria, M_i, are simply the intrinsic growth rates for this model. The Y_{oi} are the equilibria of the two species when present together (found by setting $\frac{dN_1}{dt} = 0$ and $\frac{dN_2}{dt} = 0$ and solving the resulting set of equations for N_1 and N_2). The RY_{ei} are the expected relative yields of the two species, which are simply the initial proportions of each species (here 0.5 since there are two species). The RY_{oi} are the observed relative yields of the two species: the observed yields Y_{oi} divided by the monoculture yields M_i. Finally, the ΔRY_i are the deviations from the expected relative yields: $RY_{oi} - RY_{ei}$. Plugging the terms from Equation 9 into the equations for complementarity and selection, we can express these two biodiversity effects as a function of the Lotka-Volterra parameters and the stressor effects. Here, for reasons of simplicity, we will do so for the case of $\mu_1 = \mu_2 = 1$. This represents the situation where both species have comparable fitness in absence of stress (as $\alpha_{11} = \alpha_{22}$ and $\alpha_{12} = \alpha_{21}$).

We first write the net biodiversity effect as:

$$
\begin{aligned}
\Delta Y = Y_o - Y_e &= \left(\mu_1 \prod_l^s \varepsilon_{1l} + \mu_2 \prod_l^s \varepsilon_{2l}\right)\frac{1}{1+\alpha} - \left(\mu_1 \prod_l^s \varepsilon_{1l} + \mu_2 \prod_l^s \varepsilon_{2l}\right)\frac{1}{2} \\
&= \left(\mu_1 \prod_l^s \varepsilon_{1l} + \mu_2 \prod_l^s \varepsilon_{2l}\right)\left(\frac{1}{1+\alpha} - \frac{1}{2}\right)
\end{aligned}
\tag{10}
$$

We then continue by writing complementarity C as:

$$
C = n\overline{\Delta RY} \cdot \overline{M} = \left(\frac{1}{1-\alpha^2}\left(2 - \alpha\frac{\prod_l^s \varepsilon_{2l}}{\prod_l^s \varepsilon_{1l}} - \alpha\frac{\prod_l^s \varepsilon_{1l}}{\prod_l^s \varepsilon_{2l}}\right) - 1\right)\frac{\prod_l^s \varepsilon_{1l} + \prod_l^s \varepsilon_{2l}}{2} \tag{11}
$$

Complementarity scaled by ΔY then reduces to:

$$
\frac{C}{\Delta Y} = \frac{\alpha\left(\alpha - \rho - \frac{1}{\rho}\right) + 1}{(\alpha - 1)^2} \tag{12}
$$

We can now write the selection effect S scaled by ΔY as:

$$
\frac{S}{\Delta Y} = 1 - \frac{C}{\Delta Y} = \frac{\alpha\left(\frac{1}{\rho} - 2 + \rho\right)}{(\alpha - 1)^2} \tag{13}
$$

Equations 12 and 13 allow us to understand the results for the stressor effects on complementarity and selection.

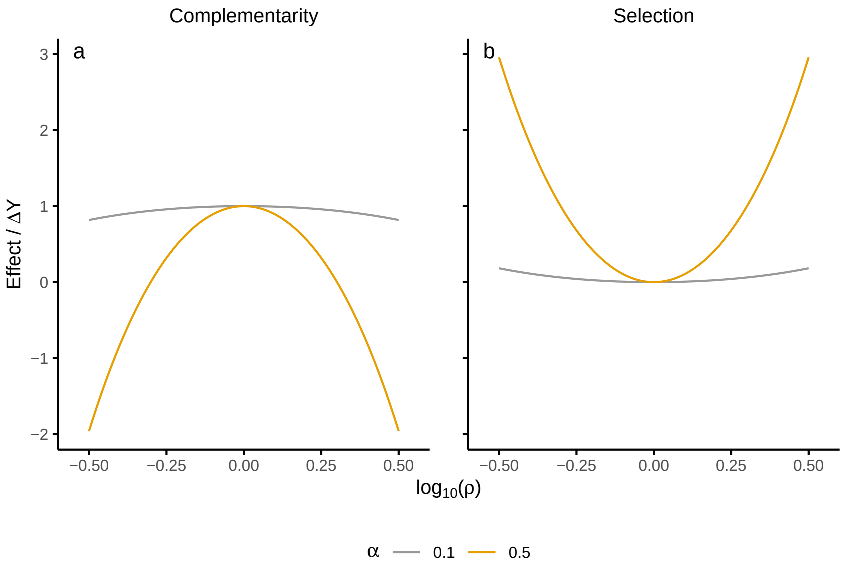

Figure S2: Equations 12 and 13 predict the change of the complementarity and selection effect, scaled by the net biodiversity effect ΔY, with the ratio of total stressor effects ρ. These changes are weaker for weak ($\alpha = 0.1$) than for strong interactions ($\alpha = 0.5$).

When average stressor intensity is unfixed, stressor richness makes the ratio ρ deviate more frequently from 1 (Figure S1). Equations 12 and 13 (visualised in Figure S2) show that greater deviations from 1 lead to lower complementarity and higher selection. Conversely, when controlling for average stressor intensity, stressor richness leads to smaller deviations from $\rho = 1$ (Figure S1), leading to higher complementarity and lower selection Figure S2). These changes are more pronounced when species interactions are stronger.

Full factorial results

Presented in the main article are only some of the combinations of the full factorial analysis, the results of all combinations are shown here, with results without stressor interactions in Figure S4 and with stressor interactions in Figure S5. Columns indicate the intensity at which stressor effects were controlled.

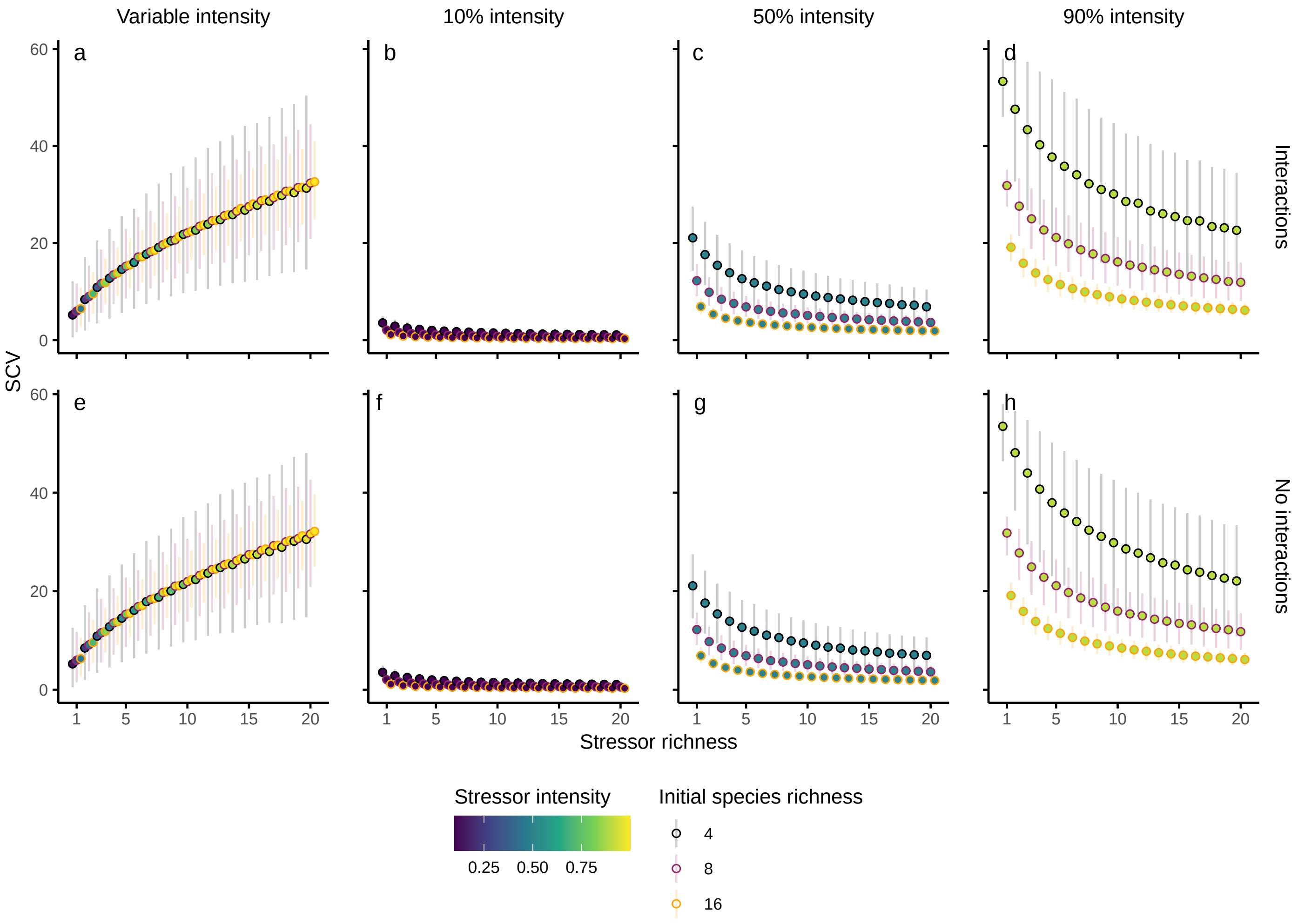

Figure S3: Effect of stressor richness on stressor coefficient of variation (SCV). Increasing initial species richness changed the point at which stressor diversity declined sharply. With increasing initial species richness, stressor diversity increases. Increasing stressor richness decreases stressor diversity, as, in larger matrices, it is less likely to resemble the identity matrix. SCV at higher species richnesses was slightly higher when stressor action was uncontrolled.

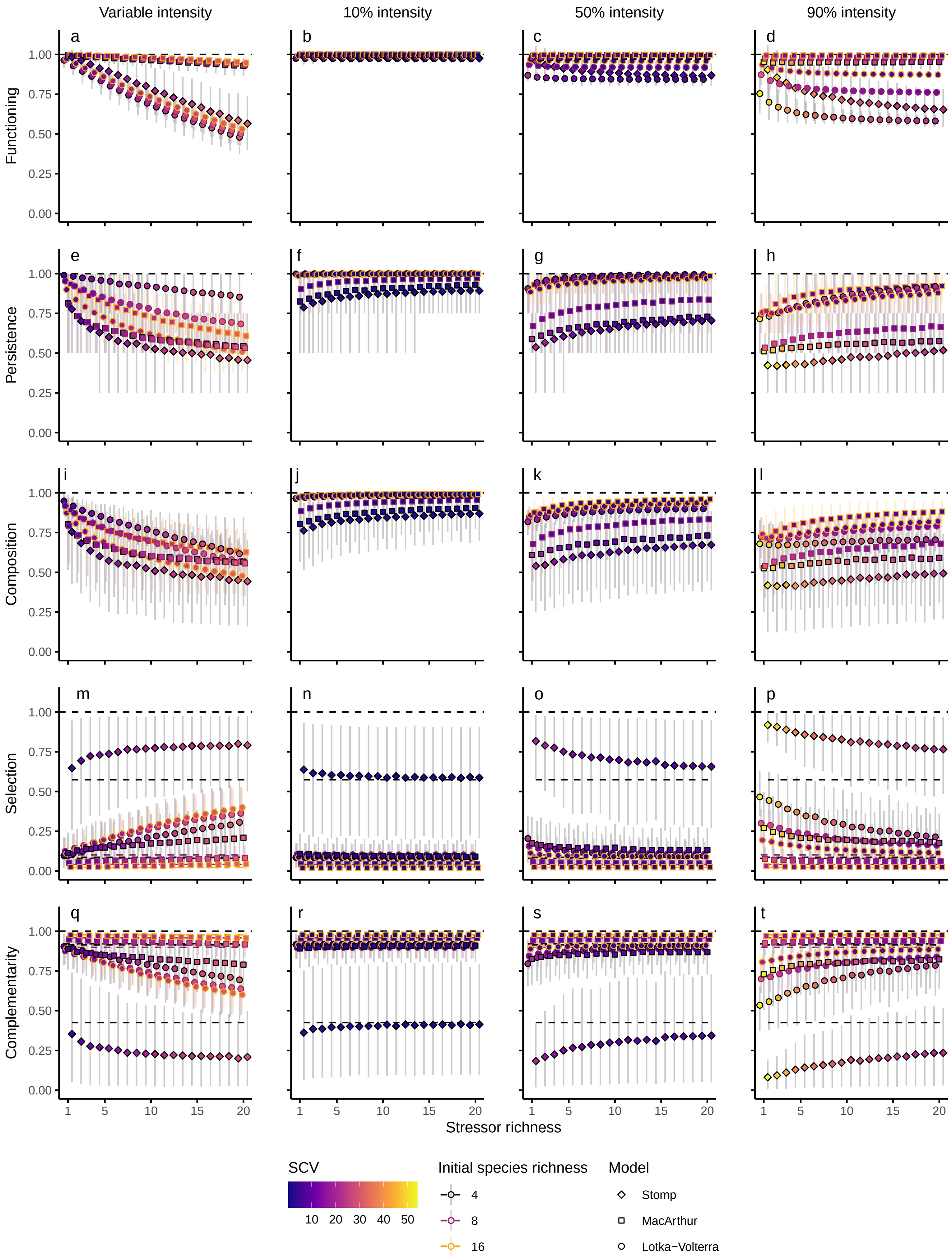

Figure S4: Non-interactive stressor effects on community and biodiversity metrics.

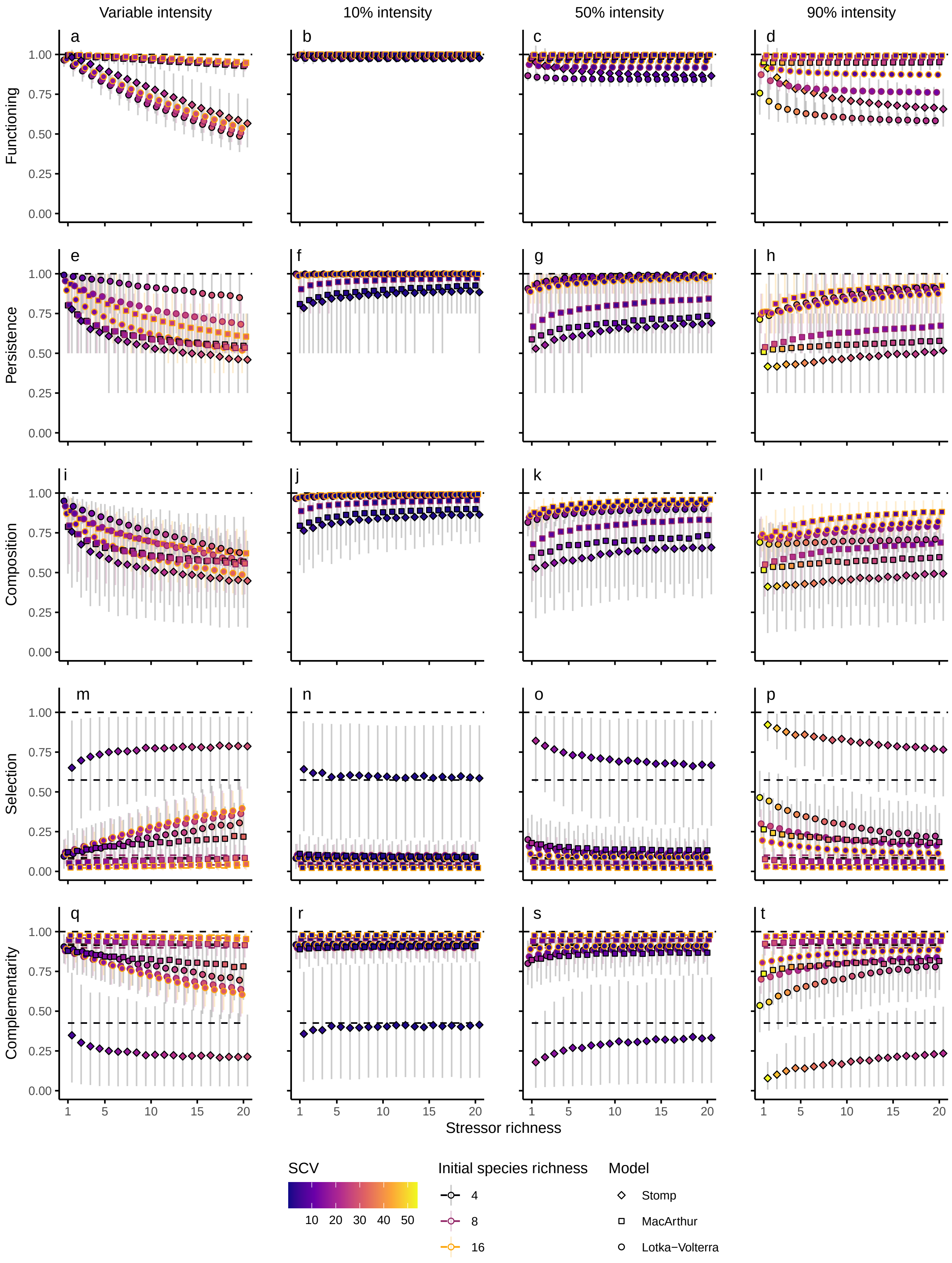

Figure S5: Interactive stressor effects on community and biodiversity metrics.

Stressor interactions

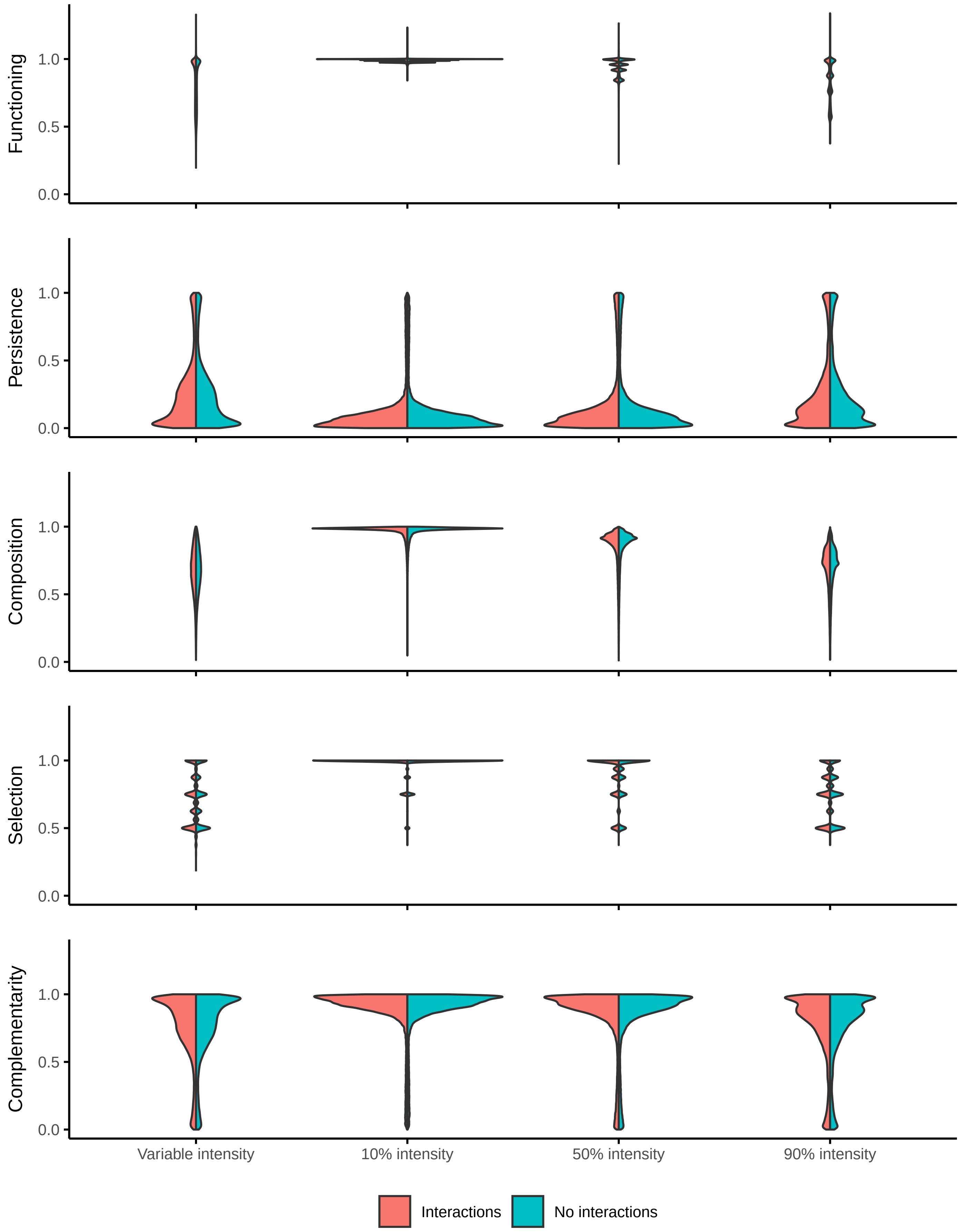

Figure S6: Effect of stressor interactions on community and biodiversity metrics

References

MacArthur, R. (1970). Species packing and competitive equilibrium for many species. *Theoretical Population Biology*, 1(1):1–11.

Spaak, J. W. and De Laender, F. (2021). Effects of pigment richness and size variation on coexistence, richness and function in light-limited phytoplankton. *Journal of Ecology*, 109(6):2385–2394.

Stomp, M., Huisman, J., De Jongh, F., Veraart, A. J., Gerla, D., Rijkeboer, M., Ibelings, B. W., Wollenzien, U. I., and Stal, L. J. (2004). Adaptive divergence in pigment composition promotes phytoplankton biodiversity. *Nature*, 432(7013):104–107.

Stomp, M., Huisman, J., Stal, L. J., and Matthijs, H. C. (2007). Colorful niches of phototrophic microorganisms shaped by vibrations of the water molecule. *ISME Journal*, 1(4):271–282.
